# Between‐year weather differences and long‐term environmental trends both contribute to observed vegetation changes in a plot resurvey study

**DOI:** 10.1002/ece3.70244

**Published:** 2024-09-02

**Authors:** László Erdős, Gábor Ónodi, Khanh Vu Ho, Eszter Tanács, Rabuogi Quinter Akinyi, Péter Török, Csaba Tölgyesi, Zoltán Bátori, György Kröel‐Dulay

**Affiliations:** ^1^ HUN‐REN Centre for Ecological Research Institute of Ecology and Botany Vácrátót Hungary; ^2^ HUN‐REN‐DE Functional and Restoration Ecology Research Group Debrecen Hungary; ^3^ Department of Ecology University of Debrecen Debrcen Hungary; ^4^ Doctoral School of Environmental Sciences University of Szeged Szeged Hungary; ^5^ Faculty of Natural Resources‐Environment Kien Giang University Kien Giang Vietnam; ^6^ Department of Plant Systematics, Ecology and Theoretical Biology Eötvös Loránd University Budapest Hungary; ^7^ Center for Environmental Science, Faculty of Science Eötvös Loránd University Budapest Hungary; ^8^ Polish Academy of Sciences Botanical Garden–Center for Biological Diversity Conservation in Powsin Warszawa Poland; ^9^ MTA‐SZTE ‘Momentum’ Applied Ecology Research Group University of Szeged Szeged Hungary; ^10^ Department of Ecology University of Szeged Szeged Hungary; ^11^ National Laboratory for Health Security HUN‐REN Centre for Ecological Research Budapest Hungary

**Keywords:** ecological indicator values, global change, interannual fluctuations, plot resurvey, quasi‐permanent plots, steppe

## Abstract

Repeated surveys of vegetation plots offer a viable tool to detect fine‐scale responses of vegetation to environmental changes. In this study, our aim was to explore how the species composition and species richness of dry grasslands changed over a period of 17 years, how these changes relate to environmental changes and how the presence of spring ephemerals, which may react to short‐term weather fluctuations rather than long‐term climatic trends, may influence the results. A total of 95 plots was surveyed in 2005 and resurveyed in 2022 in dry grasslands of the Kiskunság Sand Ridge (Hungary, Eastern Central Europe), where there has been a significant increase in mean annual temperature during the last decades, while no trends in precipitation can be identified. Db‐RDA was performed to reveal compositional changes. The changes in environmental conditions and naturalness state were assessed using ecological and naturalness indicator values. We also compared per‐plot richness of all species, native species and non‐native species of the old and the new relevés. All analyses were repeated after removing all spring ephemerals. We found clear temporal changes in species composition. Mean temperature indicator values increased, while mean soil moisture indicator values decreased during the 17 years. Also, decreasing per‐plot richness was detected both for all species and for native species, while mean naturalness increased. After the removal of spring ephemerals, the compositional changes were less obvious although still significant. The increase in the temperature indicator values remained significant even without the spring ephemerals. However, the decrease in the moisture indicator values, the decrease in the number of all species and native species, as well as the increase in naturalness indicator values disappeared when spring ephemerals were excluded from the analyses. Our study demonstrates that between‐year weather differences and long‐term environmental trends both contribute to observed vegetation changes.

## Graphical abstract

A plot resurvey spanning 17 years shows that vegetation is already reacting to the increased temperature, as indicated by the altered species composition and the significant increase in mean temperature indicator values. However, some of the detected changes, including modifications in species richness and mean indicator values for moisture, are the result of weather fluctuations rather than a long‐term climatic trend.

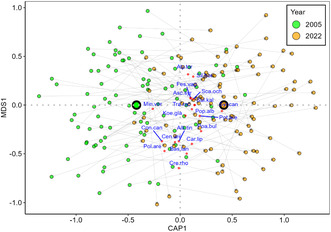

## 1 INTRODUCTION

Human activity has led to unprecedented environmental changes at multiple scales (IPBES, 2019; Millennium Ecosystem Assessment, 2005). At the global scale, the surface temperature of the Earth has increased by ca. 1.1°C compared to 1850–1900 levels and the global water cycle has also suffered major changes (IPCC, 2023). These processes are usually exacerbated by regional and local phenomena such as afforestation, irrigation, fragmentation or mining (Török et al., 2017; Meier et al., 2021; Tölgyesi et al., 2023). Corresponding vegetation changes are highly variable and may include, among others, altered species composition (e.g. Liu et al., 2018; Munson et al., 2012), modifications in plant cover and biomass (e.g. Kovács‐Láng et al., 2000; Lohmann et al., 2012), altered species diversity (e.g. Suggitt et al., 2019; Thuiller et al., 2005) or shifts of complete vegetation belts (e.g. Hanewinkel et al., 2013; Xu et al., 2009). The vegetation of arid and semi‐arid regions is thought to be especially sensitive to environmental changes, as they are already severely stressed (Almalki et al., 2022; De Keersmaecker et al., 2015).

Repeated surveys of vegetation plots offer a viable tool to detect fine‐scale responses of vegetation to environmental changes (Kapfer et al., 2017). The method has been effectively used to track decadal changes in plant communities in arctic environments (e.g. Wilson & Nilsson, 2009), temperate grasslands (e.g. Meier et al., 2021; Tölgyesi et al., 2016) and forests (e.g. Wrońska‐Pilarek et al., 2023). However, plot resurveys entail potential pitfalls. For example, observer error may occur when old and new relevés have been made by different researchers (Morrison, 2016; Verheyen et al., 2018). Relocation error happens when plots are not permanently marked and thus old and new plots are not in the same position (Kopecký & Macek, 2015; Verheyen et al., 2018). A third potential error, which has received relatively little attention, arises as a result of interannual fluctuations and associated between‐year differences in species composition between the year of the original survey and that of the resurvey (Kapfer et al., 2017).

Dry and semi‐dry grasslands in Europe have exceptional conservation importance as they host high biodiversity, contain many endemic and threatened species and provide several ecosystem services (e.g. Dengler et al., 2012; Roleček et al., 2014; Török et al., 2020; Wilson et al., 2012). While most of these grasslands are of secondary origin (i.e. anthropogenically created) in western and northern Europe, many are natural (i.e. were created by natural forces such as drought or fire) in the eastern and southeastern parts of the continent, including the eastern part of the Carpathian Basin (Török et al., 2017).

The Kiskunság Sand Ridge is a lowland area between the Rivers Danube and Tisza in Hungary (Carpathian Basin, Eastern Central Europe). Situated at the interface of the temperate deciduous forest and the forest–steppe biomes (Erdős, Ambarlı, et al., 2018), the Kiskunság is regarded as especially sensitive to aridification (Ladányi et al., 2015). Indeed, simulations showed that, due to climate change, the forest–steppe vegetation of the Kiskunság may be replaced by open steppes in the long run (Hickler et al., 2012). The situation is made worse by afforestation (primarily with the non‐native species *Pinus nigra*, *P. sylvestris* and *Robinia pseudoacacia*), irrigation and the presence of drainage canals, which, by depleting groundwater sources, have contributed significantly to the water shortage of the region (Szalai, 2011; Tölgyesi et al., 2023), resulting in a severe decrease in the groundwater level (Szalai, 2011; Zsákovics et al., 2007).

Forest–steppes, in general, and the Kiskunság Sand Ridge, in particular, are characterised by strong interannual fluctuations in precipitation (Chibilyov, 2002; Kun, 2001; Tölgyesi et al., 2016). Also, sand grasslands of the region typically host several spring ephemerals, that is, short‐lived annuals that complete their life cycle during a relatively short time (Borhidi et al., 2012). It has been shown that these species are more sensitive to interannual climate variability than longer‐lived and more abundant species (Cleland et al., 2013; Hochstrasser et al., 2002). These facts make the evaluation of plot resurvey results particularly difficult, potentially resulting in erroneous conclusions, as the interannual weather fluctuations and the resulting changes in the frequency or abundance of ephemeral species may confuse or obscure long‐term vegetation trends.

Our aim in this work was to study how species composition and species richness changed over a period of 17 years, between 2005 and 2022, using quasi‐permanent plots (i.e. plots not permanently marked but relocated with ±3 m accuracy, Kapfer et al., 2017), and using species‐based indicator values, examine how observed changes in species composition and species richness relate to environmental changes. Our second goal was to study how the presence or absence of spring ephemerals might influence the results of plot resurveys.

## 2 MATERIALS AND METHODS

### 2.1 Study region

**FIGURE 1:**
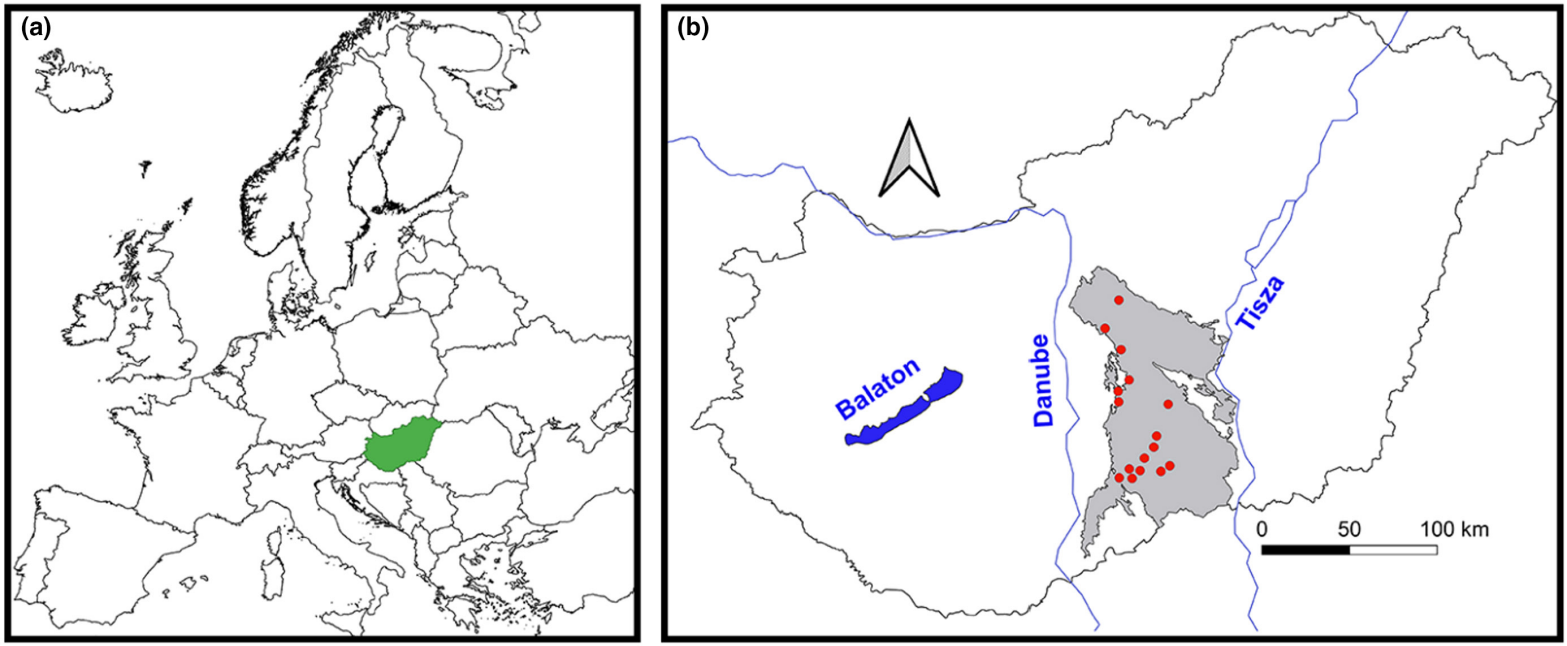
(a) The position of Hungary in Europe. (b) Study sites (red dots) in the Kiskunság Sand Ridge (grey area) between the Rivers Danube and Tisza.

The study was conducted in the Kiskunság Sand Ridge in Central Hungary (Figure 1). The climate is sub‐continental with sub‐Mediterranean influences. The mean annual temperature varies between 11 and 11.4°C across the sites, while the mean annual precipitation ranges between 545 and 602 mm (1990–2020, data from the Hungarian Meteorological Service). The region is characterised by calcareous sand dunes; soils are humus‐poor sandy soils with low water retention capacity (Várallyay, 1993).

The natural vegetation of the region is forest–steppe, that is, a mosaic of forest and grassland patches (Erdős et al., 2023), but the current landscape is dominated by agricultural areas and tree plantations. This study focused on the open perennial grassland *Festucetum vaginatae*, the most common natural grassland of the region (within the EU priority habitat type 6260 Pannonic sand steppes; alliance *Festucion vaginatae*). The overall cover of vascular plants varies between 40 and 70%. *Festuca vaginata* and *Stipa borysthenica* are the dominant species, while other frequent and abundant species include *Alkanna tinctoria*, *Euphorbia seguieriana*, *Fumana procumbens* and *Koeleria glauca*. Mosses, lichens and bare sand typically occur in the gaps among vascular species.

### 2.2 Study sites and field sampling

Sixteen sites, 1 km × 1 km each, were selected for the study (Figure 1b, Table 1). Site selection was based on the following criteria: (a) all major remaining sand dune areas were to be covered (excluding an actively used military training ground), (b) near‐natural sand forest–steppe (including grasslands and woodlands) was to make up at least 20% of the site and (c) neighbouring sites were to be at least 5 km from each other and to be separated by areas where other land‐use types dominate (typically agricultural land or tree plantation).

**TABLE 1:** Coordinates of the study sites.

Site	Coordinates	
Balotaszállás	N46° 23′ 29.30”	E19° 33′ 03.01”
Császártöltés	N46° 23′ 09.01”	E19° 14′ 18.00”
Csévharaszt	N47° 17′ 22.98”	E19° 23′ 46.39”
Felső‐Tázlár	N46° 34′ 27.05”	E19° 33′ 06.37”
Fülöpháza	N46° 52′ 38.09”	E19° 23′ 57.53”
Imrehegy	N46° 25′ 28.61”	E19° 19′ 13.30”
Jakabszállás	N46° 43′ 41.04”	E19° 40′ 01.04”
Kéleshalom	N46° 22′ 28.82”	E19° 19′ 57.64”
Kisizsák	N46° 49′ 36.16”	E19° 18′ 27.75”
Kunfehértó	N46° 24′ 33.07”	E19° 23′ 55.02”
Kunpeszér	N47° 09′ 16.76”	E19° 16′ 01.00”
Pirtó	N46° 28′ 10.32”	E19° 26′ 29.93”
Sarlósár	N47° 02′ 10.71”	E19° 22′ 00.73”
Soltszentimre	N46° 46′ 20.79”	E19° 18′ 08.47”
Tázlár	N46° 31′ 09.97”	E19° 31′ 15.08”
Zsana	N46° 24′ 50.47”	E19° 37′ 19.05”

**FIGURE 2:**
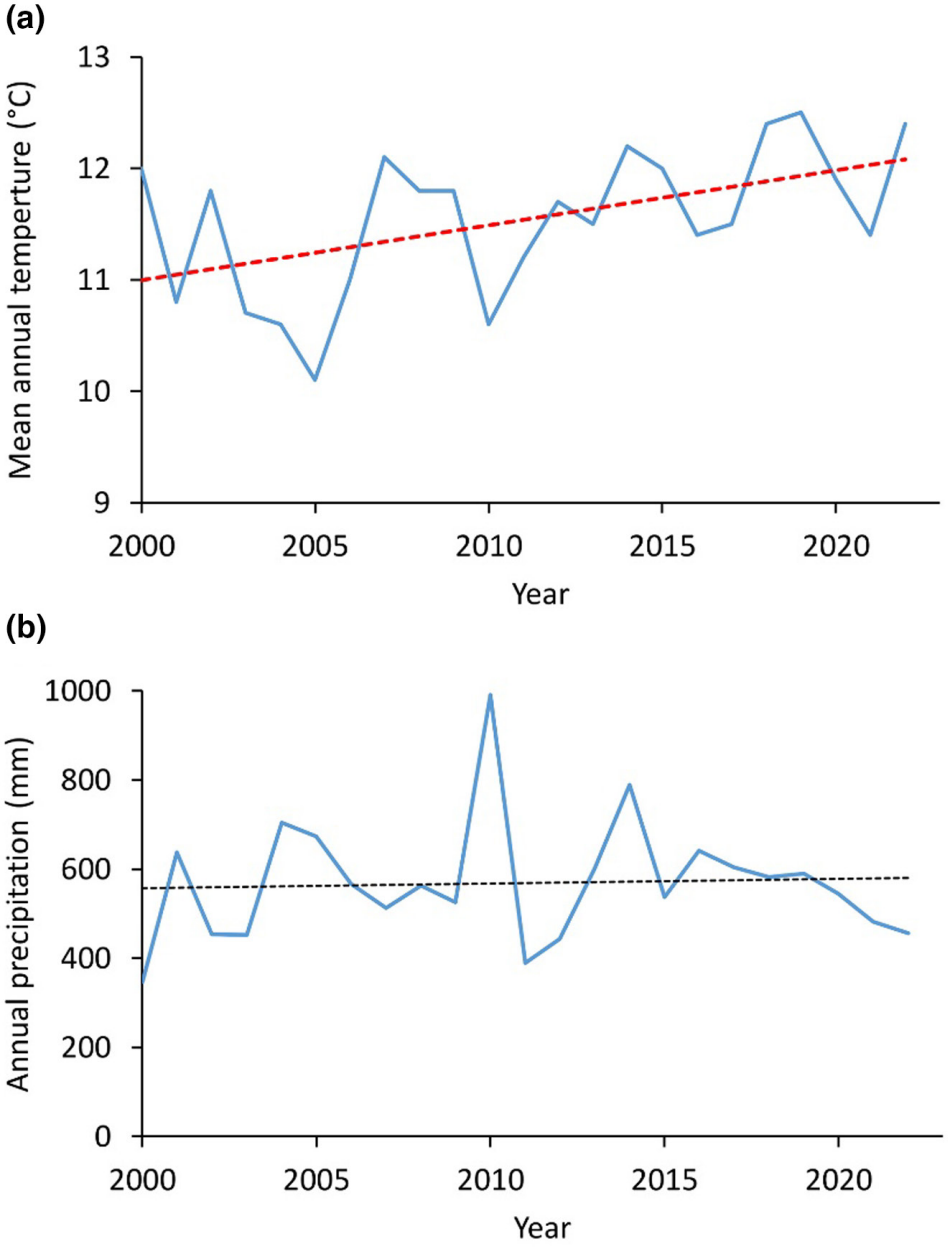
(a) Changes in mean annual temperature across the 16 study sites between 2000 and 2022 (adjusted *R* ^2^ = .226, *F* = 7.413, *p* = .013, intercept: −87.1 and slope: 0.05). (b) Changes in annual precipitation across the 16 study sites between 2000 and 2022 (adjusted *R* ^2^ = −0.045, *F* = 0.057, *p* = .814, intercept: −1610 and slope: 1.08). Data are from the Hungarian Meteorological Survey.

The sites have experienced considerable increases in mean annual temperature between 2000 and 2022, while no clear trend is visible regarding annual precipitation (Figure 2).

Within each study site, six sampling plots, 4 m × 4 m each, were selected in 2005. During plot selection, spatially stratified random sampling was applied: We kept a minimum distance between neighbouring sampling points because we wanted to avoid the sampling points that were too close to each other and were thus spatially autocorrelated. We generated a random point, but it was only accepted for sampling in the field if it was located in open perennial sand grassland and it was at least 140 m from an already selected sampling plot. We then checked the next randomly generated point, applied the same criteria and continued this algorithm as long as we reached six sampling points at a site. Point coordinates were archived and used for resampling in 2022. Points in 2005 were not marked permanently in the field, but we used a GPS device to relocate them in 2022. Since the accuracy of the device is 2–3 m (based on experience from the fieldworks with permanently marked field points) and plot size is 4 m × 4 m, the plots can be considered quasi‐permanent (Kapfer et al., 2017).

In each plot, we listed all vascular plants present and visually estimated percentage cover by species in both years. Sampling was performed at the end of spring and the beginning of summer when both spring and summer species were present. Please note that in this study, we only use presence/absence data. In both years, 2005 and 2022, the same scientist led the fieldwork (G. Kröel‐Dulay). In one site, one sampling plot was converted to tree plantations, so for this site we only resampled five plots, resulting in a total of 95 plots (15 × 6 + 5 = 95), where there were no signs of anthropogenic interventions.

### 2.3 Data analyses

To reveal the change in species composition between the two different years, we applied partial distance‐based redundancy analysis (db‐RDA) using Sørensen‐Dice dissimilarity on species presence–absence data (function: capscale). Community composition was used as the response variable, year as the predictor and plot identity and site as conditional predictors. To test whether time had a significant effect on species composition, a permutational multivariate analysis of variance (PERMANOVA) with 999 permutations was applied using ‘anova’ function. All mentioned functions were available in the vegan package of R version 4.3.2 (Oksanen et al., 2022; R Core Team, 2023).

We used ecological indicator values to interpret changes in environmental conditions over time. Ecological indicator values are highly correlated to field measurements regarding soil conditions and climate factors (Diekmann et al., 1999; Ertsen et al., 1998; Pröll et al., 2011; Schaffers & Sýkora, 2000). In addition, ecological indicator values consider the fluctuating nature of environmental conditions over time (instead of providing data only for a short period), while also reflecting site conditions in accordance with species requirements (Diekmann, 2003; van der Maarel, 1993; Zonneveld, 1983). For this study, we computed the unweighted (i.e. not weighted by cover) mean ecological indicator values for temperature (*T*‐value), soil moisture (*W*‐value) and nutrient supply (*N*‐value) per plot. Ellenberg (1979), Kowarik and Seidling (1989) and Ellenberg et al. (1992) recommended the use of unweighted mean ecological indicator values because the abundance of a given species in a plot reflects not only the environmental factors but also the inherent biological characteristics of the species (including its size or its ability to spread clonally). Also, when actual instrumental measurements were compared with abundance‐weighted and unweighted mean ecological indicator values, unweighted means were shown to be more reliable than cover‐weighted means (e.g. Carpenter & Goodenough, 2014; Diekmann, 1995), although the difference between unweighted and cover‐weighted means is usually small (Diekmann, 2003). We used the ecological indicator values of Borhidi (1995), which are based on the values of Ellenberg et al. (1992) and extended to the Carpathian Basin. Indicator values were retrieved from the PADAPT database (Sonkoly et al., 2023).

We calculated the per‐plot richness of all species. In addition, we also counted the number of native and non‐native species per plot. Finally, we assessed the naturalness of the grasslands in both years, based on the naturalness indicator values of Borhidi (1995). The approach is similar to the ecological indicator values and has been increasingly applied in the Carpathian Basin (e.g. Erdős et al., 2017; Erdős, Kröel‐Dulay, et al., 2018; Erdős, Török, et al., 2022; Ho et al., 2023). The method rests on the observation that different plant species have different tolerances for degradation (e.g. some plants favour natural habitats, while others are likely to tolerate or even benefit from degradation; Erdős, Bede‐Fazekas, et al., 2022; Erdős, Török, et al., 2022). Species related to natural habitats have high scores, while species associated with degraded areas receive low scores. We calculated the unweighted mean naturalness value for each plot.

We analysed the plot‐level mean ecological indicator values (*T*, *W* and *N*), as well as the per‐plot number of all species, native species and mean naturalness values with linear mixed‐effects models. However, we used a zero‐inflated linear mixed‐effect model for the per‐plot number of non‐native species, as the data had an excess of zero values. The fixed factor was the year, while the random factor was replication nested within site. We used the ‘glmmTMB’ function of the glmmTMB package to create the models with the Poisson family for count data and Gaussian family for continuous data (Brooks et al., 2017). The models were checked by visual assessments of diagnostic plots, using the ‘check_model’ function of the performance package (Lüdecke et al., 2021). Finally, we used the ‘summary’ function in R on these models to reveal the difference between the 2 years.

To study how the presence of spring ephemerals may influence the results, we removed all spring ephemerals from the data set and repeated all the above analyses with these modified data. Spring ephemerals were defined as species that have therophyte (i.e. annual) life history and start to flower during the spring months (i.e. from March to May). Life history and flowering time data were retrieved from the PADAPT database (Sonkoly et al., 2023). The following species were excluded from the second set of analyses: *Acinos arvensis*, *Alyssum alyssoides*, *Arenaria serpyllifolia*, *Bromus squarrosus*, *B. tectorum*, *Cerastium semidecandrum*, *Camelina microcarpa*, *Erodium cicutarium*, *Erophyla verna*, *Holosteum umbellatum*, *Medicago minima*, *Minuartia glomerata*, *Myosotis stricta*, *Saxifraga tridactylites*, *Secale sylvestre*, *Silene conica* and *Viola kitaibeliana*.

In order to find out if there are species that are significantly concentrated in 1 year while rare or absent in the other year, we identified diagnostic species. In this study, phi‐coefficient was computed as an indicator of fidelity (Chytrý et al., 2002). We used a phi‐value of 0.2 as the minimum threshold to determine diagnostic species. Fisher's exact test was used to rule out non‐significant diagnostic species. Analyses were conducted using the software JUICE 7.1.30 (Tichý, 2002).

## 3 RESULTS

### 3.1 Analyses with all species included

**FIGURE 3:**
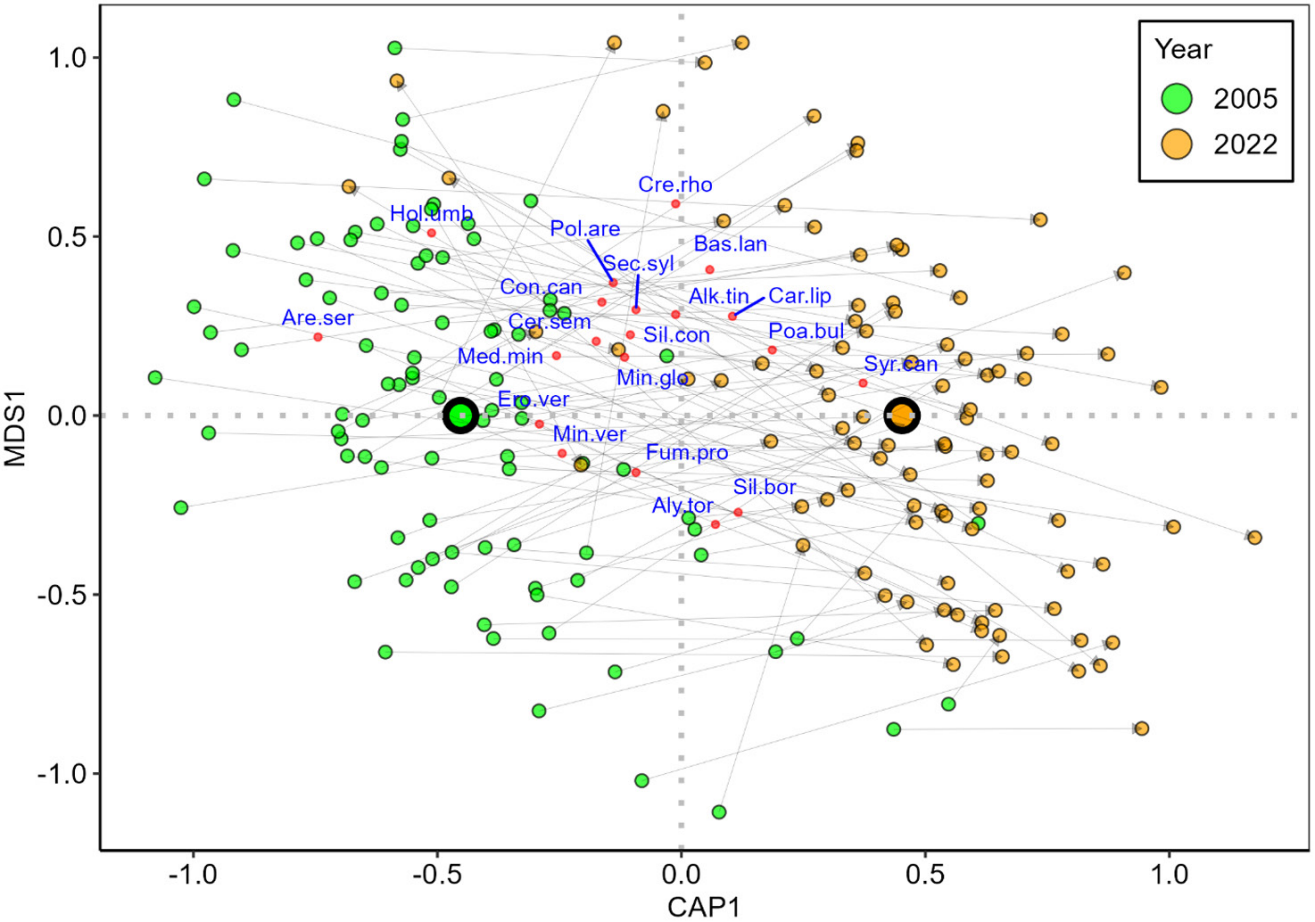
Ordination plot of the partial distance‐based redundancy analysis (db‐RDA), using all species recorded in the relevés. Pairs of old and new relevés are indicated by grey arrows. Large symbols indicate the centroids for each year. Only the top 20 species, according to the correlation to the ordination space (i.e. square root of the goodness of fit), are displayed. Spring ephemerals are highlighted in bold. Species codes are as follows: Alk.tin, *Alkanna tinctoria*; Aly.tor, *Alyssum tortuosum*; Are.ser, ***Arenaria serpyllyfolia***; Bas.lan, *Bassia laniflora*; Car.lip, *Carex liparicarpos*; Cer.sem, ***Cerastium semidecandrum***; Con.can, *Conyza canadensis*; Cre.rho, *Crepis rhoedifolia*; Ero.ver, ***Erophyla verna***; Fum.pro, *Fumana procumbens*; Hol.umb, ***Holosteum umbellatum***; Med.min, ***Medicago minima***; Min.glo, ***Minuartia glomerata***; Min.ver, *Minuartia verna*; Poa.bul, *Poa bulbosa*; Pol.are, *Polygonum arenarium*; Sec.syl, ***Secale sylvestre***; Sil.bor, *Silene borysthenica*; Sil.con, ***Silene conica***; Syr.can, *Syrenia cana*.

There was a clear separation between the old and the new relevés in the db‐RDA ordination space (Figure 3), and the PERMANOVA indicated that time had a highly significant effect on species composition (*F* = 9.615, *p* < .001). The position of the old and new relevés indicated clear and mostly consistent temporal changes in species composition, proceeding from the left to the right area of the ordination space for the overwhelming majority of the relevé pairs. There were several spring ephemerals among the most important species in the ordination space (e.g. *Arenaria serpyllyfolia* and *Holosteum umbellatum*).

**FIGURE 4:**
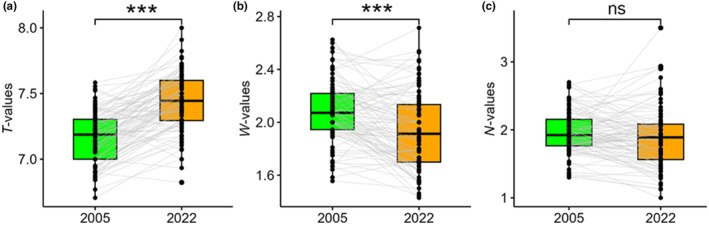
Mean ecological indicator values for (a) temperature (*T*‐values), (b) soil moisture (*W*‐values) and (c) nutrient supply (*N*‐values) between the old (2005) and new (2022) relevés for data with all species. ***: *p* < .001; ns: *p* > .05.

The old relevés had significantly lower mean ecological indicator values for temperature than new ones (*z* = −11.8, *p* < .001) (Figure 4a), but the opposite pattern was found for soil moisture (*z* = 5.22, *p* < .001) (Figure 4b). Mean ecological indicators for nutrient supply did not differ significantly between the old and the new relevés (Figure 4c).

**FIGURE 5:**
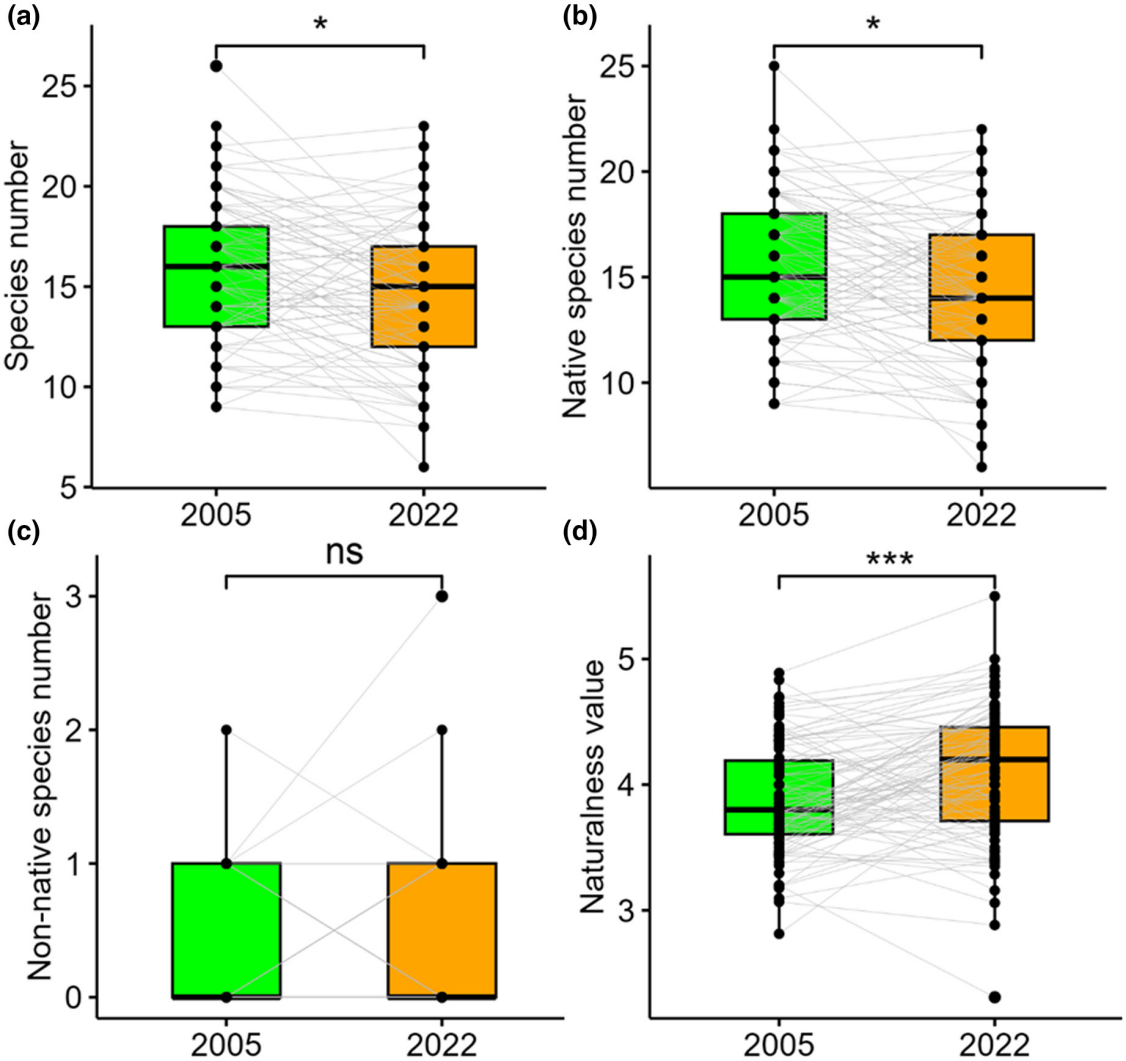
The per‐plot number of all species (a), native species (b), non‐native species (c) and mean naturalness values (d) of the old (2005) and new (2022) relevés for data with all species. ***: *p* < .001; *: *p* < .05; ns: *p* > .05.

The per‐plot number of all species was higher in the old relevés than in the new ones (*z* = 2.43, *p* = .015) (Figure 5a). A similar pattern was detected for the per‐plot number of native species (*z* = 2.48, *p* = .013) (Figure 5b). There was no significant difference in non‐native species number per plot between old and new relevés (Figure 5c). Non‐native species did not occur in the majority of the relevés, and the maximum per‐plot number of non‐native species was 3. The mean naturalness value of the old relevés was significantly lower than that of the new ones (*z* = −4.10, *p* < .001) (Figure 5d).

### 3.2 Analyses with the exclusion of spring ephemerals

**FIGURE 6:**
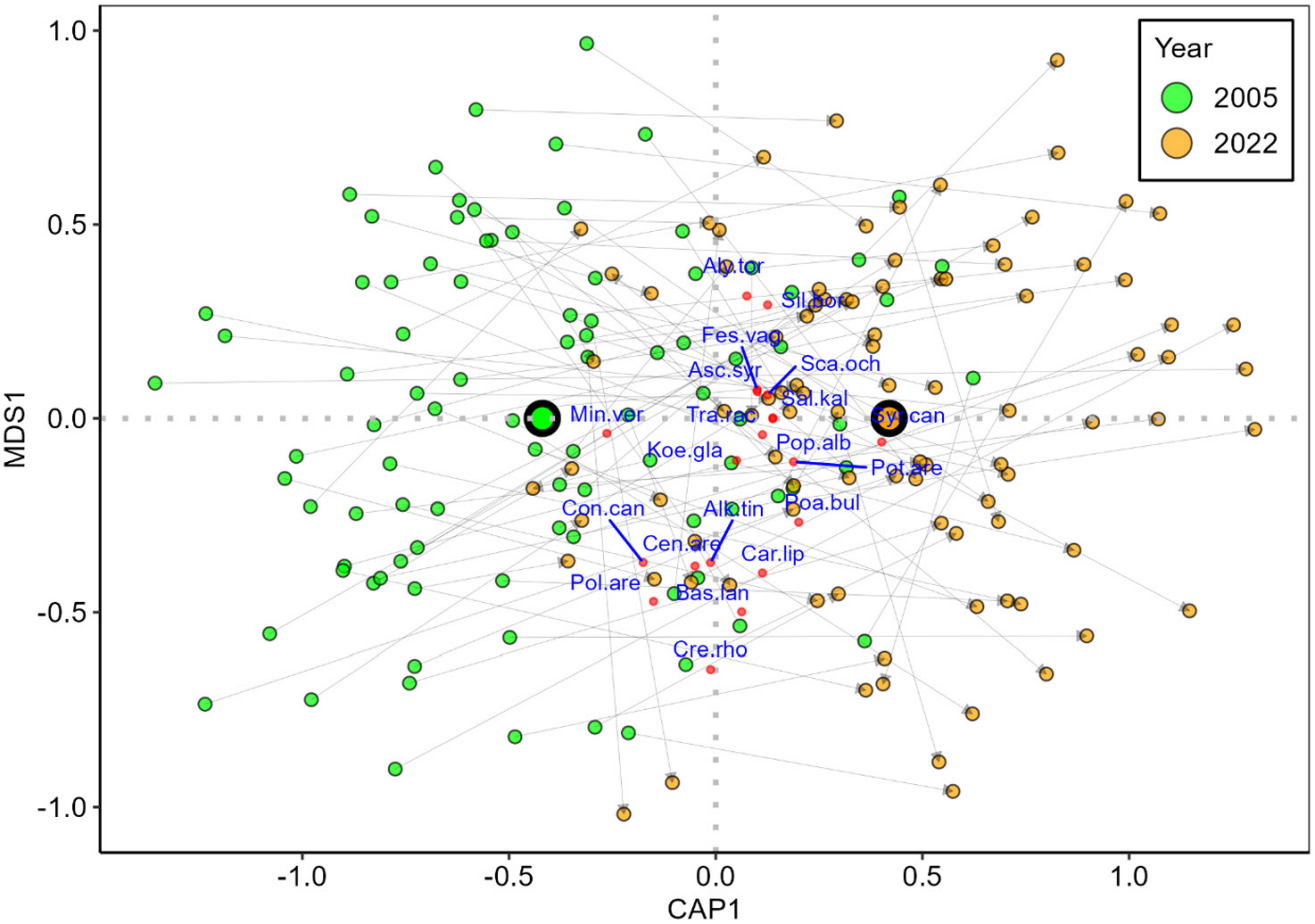
Ordination plot of partial distance‐based redundancy analysis (db‐RDA), with spring ephemerals excluded from the analysis. Pairs of old and new relevés are indicated by grey arrows. Large symbols indicate the centroids for each year. Only the top 20 species, according to the correlation to the ordination space (i.e. square root of the goodness of fit), are displayed. Species codes are as follows: Alk.tin, *Alkanna tinctoria*; Aly.tor, *Alyssum tortuosum*; Asc.syr, *Asclepias syriaca*; Bas.lan, *Bassia laniflora*; Car.lip, *Carex liparicarpos*; Cen.are, *Centaurea arenaria*; Con.can, *Conyza canadensis*; Cre.rho, *Crepis rhoedifolia*; Fes.vag, *Festuca vaginata*; Koe.gla, *Koeleria glauca*; Min.ver, *Minuartia verna*; Poa.bul, *Poa bulbosa*; Pol.are, *Polygonum arenarium*; Pop.alb, *Populus alba*; Pot.are, *Potentilla arenaria*; Sal.kal, *Salsola kali*; Sca.och, *Scabiosa ochroleuca*; Sil.bor, *Silene borysthenica*; Syr.can, *Syrenia cana*; Tra.rac, *Tragus racemosus*.

When spring ephemerals were excluded from the ordination, the separation between the old and the new relevés was less clear (Figure 6), although time still had a highly significant effect on species composition, as indicated by the PERMANOVA (*F* = 4.073, *p* < .001).

**FIGURE 7:**
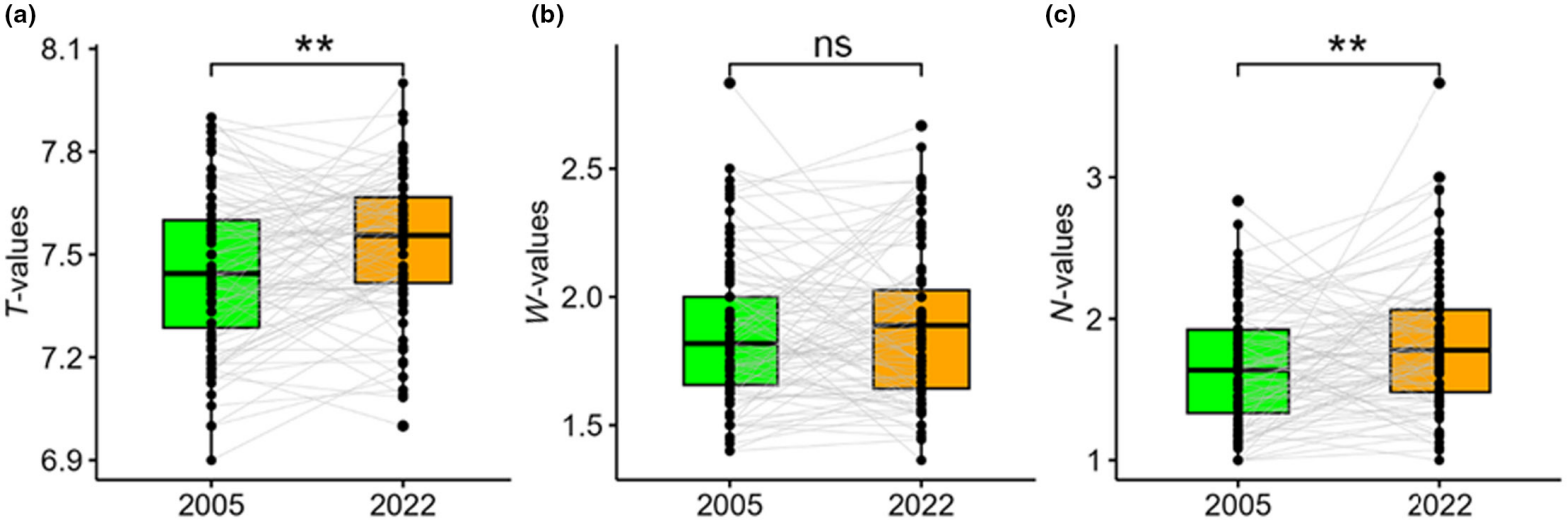
Mean ecological indicator values for (a) temperature (*T*‐values), (b) soil moisture (*W*‐values) and (c) nutrient supply (*N*‐values) between the old (2005) and new (2022) relevés after the exclusion of spring ephemerals. **: *p* < .01; ns: *p* > .05.

After the exclusion of spring ephemerals, mean indicator values for temperature were significantly lower for the old relevés than for the new ones (*z* = −2.98, *p* = .003) (Figure 7a), while no differences were found when comparing mean indicator values for soil moisture (Figure 7b). Mean indicator values for nutrient supply were lower in the old than in the new relevés (*z* = −2.95, *p* = .003) (Figure 7c).

**FIGURE 8:**
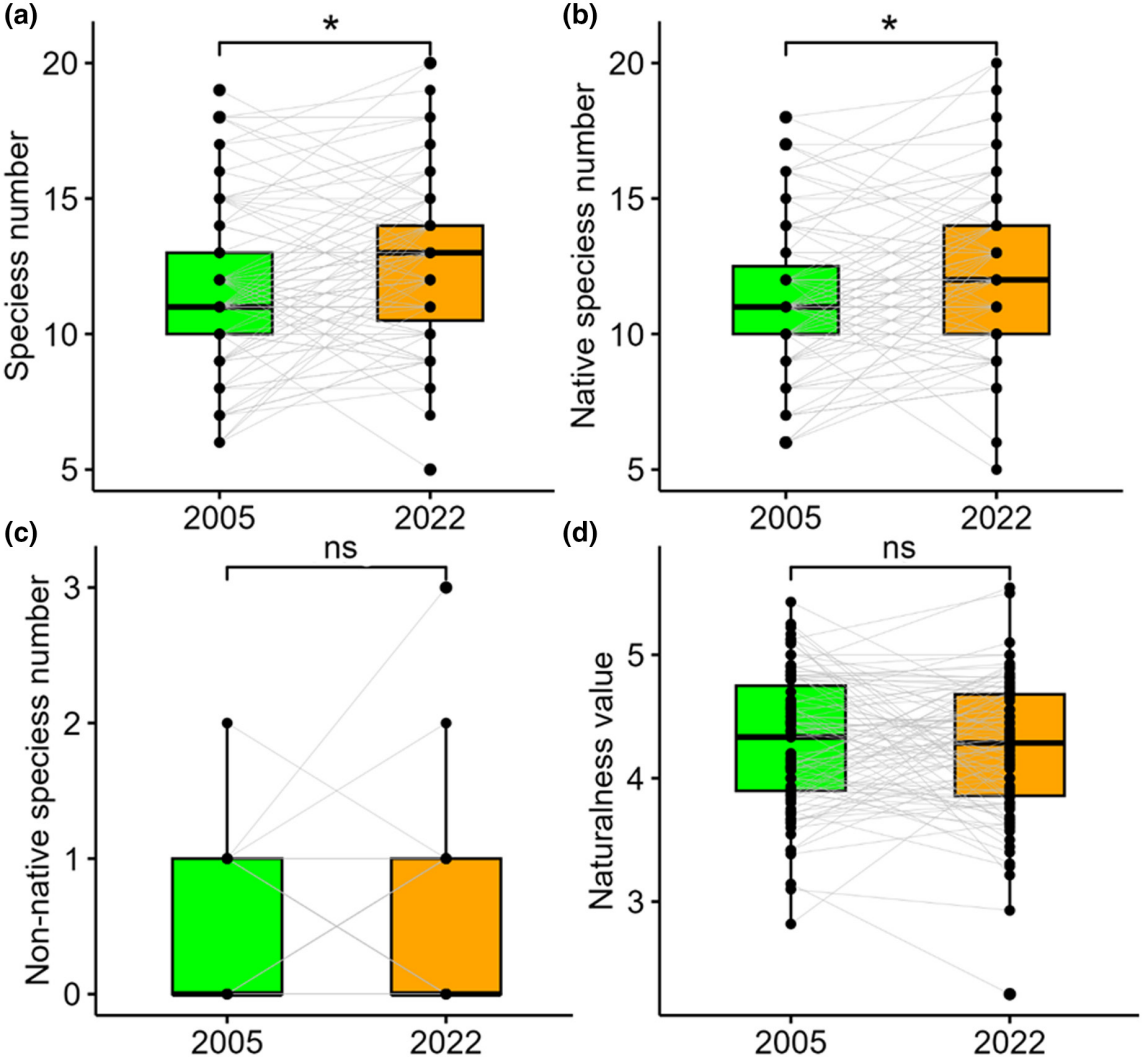
The per‐plot number of all species (a), native species (b), non‐native species (c) and mean naturalness values (d) of the old (2005) and new (2022) relevés after the exclusion of spring ephemerals. *: *p* < .05; ns: *p* > .05.

When spring ephemerals were excluded, the per‐plot number of all species was lower in the old than in the new relevés (*z* = −2.1, *p* = .035) (Figure 8a) and a similar trend was found for the number of native species (*z* = −2.11, *p* = .035) (Figure 8b). In contrast, no significant differences were found between old and new relevés for the per‐plot number of non‐native species (Figure 8c) and the mean naturalness indicators (Figure 8d).

### 3.3 Diagnostic species

Based on the fidelity values (Table 2), we found that the old relevés had eight diagnostic species, five of which were early spring ephemerals (*Arenaria serpyllifolia, Erophila verna, Holosteum umbellatum, Myosotis stricta* and *Medicago minima)*. Four species were identified as diagnostic species for new relevés.

**TABLE 2:** Diagnostic species of the old (2005) and new (2022) relevés with fidelity values (*p < .05, **p < .01, ***p < .001).

	2005	2022
***Arenaria serpyllifolia***	56.3***	
***Erophila verna***	47.1***	
***Holosteum umbellatum***	39.6***	
***Myosotis stricta***	27.8**	
*Minuartia verna*	26.4***	
*Colhicum arenarium*	22.6*	
*Stipa capillata*	22.6*	
***Medicago minima***	20.9***	
*Populus alba*		30.8***
*Syrenia cana*		27.2***
*Sporobolus cryptandrus*		24.5**
*Tragus racemosus*		22.4**

*Note*: Spring ephemerals are in bold.

## 4 DISCUSSION

In this study, using quasi‐permanent plots, we aimed to scrutinise how species composition and species richness have changed over a 17‐year period and how these relate to environmental changes. We found marked changes in species composition, species richness and ecological indicator values. However, when removing the spring ephemerals from the data (because ephemerals were thought to reflect between‐year weather differences rather than long‐term trends), some of the results changed fundamentally, indicating that short‐term fluctuations may confuse the results of plot resurveys.

Results of earlier studies using plot resurveys vary considerably (Table 3). The variability of the results in these previous studies may partly be explained by the confounding effect of factors such as landscape configuration, native animal populations or changes in land use. Also, plant communities may differ in their resistance to environmental changes.

**TABLE 3:** Selected plot resurvey studies from European dry and semi‐dry grasslands and their main findings regarding changes in species composition, per‐plot species richness (S), mean Ellenberg‐type indicators for temperature (T), soil moisture (W), nutrient supply (N) and light availability (L).

Study system	Region	Old relevés	Recent relevés	Study period (years)	Changes in						Reference
					Composition	S	T	W	N	L	
Festuco‐Brometea, Koelerio‐Corynephoretea	Central Germany	1995–2002	2018–2019	≤24	1	→					Meier et al. (2021)
Festuco‐Brometea	Central Germany	1995	2019	24	2	↓	↑	→	→	↑	Meier et al. (2022)
Festuco‐Brometea, Koelerio‐Corynephoretea	NE Germany	1993–1997	2015	≤22	1	↑	→	→	→	→	Hüllbusch et al. (2016)
Koelerio‐Corynephoretea, Sedo‐Scleranthetea	NE Germany	1994	2019	25	1	↑		↑	↑	↓	Schüle et al. (2023)
Calcareous grassland	SW England	1970	2016	46	2	↓					Ridding et al. (2020)
Calcareous grassland	Scotland	1958–1987	2012–2014	≤56	2	↑		↑	↑	→	Mitchell et al. (2017)
Brometalia erecti	NE Austria	1990–1992	2011	≤21		↓			↑	↓	Hülber et al. (2017)
Festucetea vaginatae	Central Hungary	1999	2009	10	1		↑	↓	↓		Tölgyesi and Körmöczi (2012)
Mostly Festuco‐Brometea	SE Czech Republic	1985–1986	2022	≤37	2	↓	↓	↑	↑	↓	Klinkovská et al. (2024)
Calluno‐Ulicetea, Koelerio‐Corynephoretea, Festuco‐Brometea	S Czech Republic	1986–1991	2018–2019	≤33	1	↓	→	→	↑	→	Harásek et al. (2023)

*Note*: 1: Moderate compositional changes and 2: considerable compositional changes. Empty cells mean that the cited work did not measure the given characteristic.

Vegetation resurveys using quasi‐permanent plots represent a powerful tool to study temporal changes in vegetation, but results must be evaluated with caution, and efforts have to be taken to avoid potential pitfalls. In our study, we reduced observer bias by using presence–absence data instead of cover values. Thus, we were able to avoid estimation errors (mistakes that arise due to the somewhat subjective cover estimation performed by different researchers), which is a considerable source of error (Morrison et al., 2020). Resurveys are generally robust to relocation error (Kopecký & Macek, 2015), and this was further minimised by the fact that GPS coordinates were available for the old plots. Also, the rather large sample size in the current study decreased bias from relocation error (Kapfer et al., 2017; Verheyen et al., 2018). A third source of uncertainty, the interannual variability of environmental factors, has received surprisingly little attention so far in the literature. Fischer et al. (2020) showed that between‐year weather differences may have a greater effect on vegetation than long‐term environmental changes. Also, the results of Fischer et al. (2020) highlight that plants with different life histories react differently to the environmental factors of the actual year as well as the few preceding years. For example, wet summers favour perennials while wet springs favour annuals. This is in line with our field experience. In unusually dry springs, the frequency and abundance of spring ephemerals are very low in the sand grasslands of the Kiskunság (personal observation). This could be one reason why we found so few of these species in 2022. The mean precipitation of the 16 sites from 1st February to 30th April was 143 mm in 2005, but only 72 mm in 2022 (data from the Hungarian Meteorological Service). In addition, in 2005, these grasslands were still recovering from a huge drought event in 2003, which hit the perennial grasses hard and thus allowed annuals to thrive, at least in the central part of the study area (Orbán et al., 2023). A very similar effect was found in *Festucion valesiacae* grasslands of the Czech Republic (Fischer et al., 2020).

Our results demonstrate that the short‐term fluctuation of spring ephemerals as a response to weather variability may have a considerable effect on the changes detected by plot resurveys. We found clearly identifiable species compositional changes between the 2 years, but the changes were less obvious when spring ephemerals were excluded from data analysis. The significant increase in mean indicator values for temperatures, both with and without spring ephemerals, suggests that vegetation is already reacting to the increasing temperature. On the other hand, the apparent decrease in soil moisture values (when all species were analysed) disappeared when ephemerals were excluded, emphasising that fluctuations of spring ephemerals may indeed have a confounding effect on plot resurvey results. The observed changes in ecological indicator values without spring ephemerals (increasing temperature values and no change in moisture values) are in line with the observed climatic trends in the region.

Similarly, the observed decrease in per‐plot richness of all species and native species disappeared when spring ephemerals were excluded, suggesting that, as yet, no decrease in species richness is detectable in the studied grasslands at the scale used in our work.

The between‐year differences in the frequency of spring ephemerals offer an explanation of why the mean naturalness values showed a significant increase between 2005 and 2022 in the original analyses. Spring ephemerals tend to have low naturalness values (Zinnen et al., 2021), so their high frequency in 2005 resulted in low mean naturalness in that year. In 2022, when spring ephemerals were rare, mean naturalness values increased. The difference disappeared when ephemerals were excluded from the analysis, suggesting that there has been no real trend in naturalness, but probably only a short‐term fluctuation. This result indicates that similar to ecological indicator values, naturalness values have to be evaluated with caution during plot resurveys.

Our analysis of diagnostic species shows that *Populus alba* has become more frequent during the study period. Eurasian forest–steppes were grazed by large herds of native ungulates, which were replaced by domesticated animals in historical times (Erdős, Török, et al., 2022; Molnár et al., 2012). Grazing undoubtedly limited the spread of woody vegetation, shifting the woody‐herbaceous balance and enabling only a lower tree and shrub cover than would be possible climatically (Chytrý et al., 2022; Erdős, Török, et al., 2022). After the cessation of grazing in the 20th Century, woody species were released from grazing pressure, which may be the main reason behind the forest and shrub encroachment observable in some parts of the Kiskunság Sand Ridge. *Populus alba* is capable of making use of this process due to its rapid vertical growth, excellent clonal spread and tolerance to drought (Kopecky, 1978). Moreover, ramets are in contact below the soil surface through root suckers. Thus, trees growing under relatively moist circumstances (e.g. in dune slacks) can allocate resources to trees living in harsher sites (Magyar, 1961).

Sand grasslands of the region are sensitive to biological invasion (Botta‐Dukát, 2008). We believe that the appearance of *Sporobolus cryptandrus* and *Tragus racemosus* among the diagnostic species for the new relevés indicates the spread of these invasive species. Both are C4 grasses. Thus, under increasing temperatures and decreasing water availability, they are expected to gain competitive advantage over native C3 species (Török et al., 2021). *Tragus racemosus* probably originated from South Africa and appeared in Hungary hundreds of years ago (Schmidt, 2012). It is a widespread species in the Kiskunság Sand Ridge, but, as yet, seems to have little impact on the native vegetation (Schmidt, 2012). *Sporobolus cryptandrus* is native to North American prairies, deserts and chaparral communities. Its first reliable record in Hungary is from 2016 (Török et al., 2021; Török & Aradi, 2017). It mainly occurs in disturbed sites (e.g. along roads and in burned areas), but it is also able to invade intact ecosystems. It is particularly dangerous that after severe droughts, individuals of *S. cryptandrus* can replace dying bunches of *Festuca vaginata*, the dominant grass in the open sand grasslands of the Kiskunság Sand Ridge (Török et al., 2021). Recent evidence indicates that *S. cryptandrus* has serious negative effects on the invaded vegetation (Hábenczyus et al., 2022). Our results highlight the importance of invasion processes currently happening in the study region.

## 5 CONCLUSIONS

In this work, we aimed to analyse vegetation changes over a 17‐year period. Very clear and consistent temporal changes in species composition were revealed. Mean temperature values increased, while mean soil moisture values decreased during the study period. Per‐plot richness decreased for all species and also for native species. In the second set of analyses, when spring ephemerals were excluded, some of the results changed considerably. Compositional changes were less marked although still highly significant. The increase in mean temperature indicator values remained significant, while the decrease in soil moisture values disappeared. Also, the decrease in the per‐plot richness of all species and native species, as well as the increase in mean naturalness disappeared when ephemerals were removed from the analyses.

Our study indicates that between‐year weather differences and long‐term environmental trends both contribute to observed vegetation changes in plot resurvey studies. Therefore, it is necessary to carefully check the weather conditions of the study years when two points in time are compared using plot resurvey data. Using time series (i.e. comparing more than two points in time) can provide results that are easier to interpret, especially in ecosystems where interannual climate variation is considerable.

## AUTHOR CONTRIBUTIONS

**László Erdős:** Conceptualization (supporting); funding acquisition (supporting); investigation (supporting); methodology (lead); project administration (supporting); supervision (lead); writing – original draft (lead); writing – review and editing (lead). **Gábor Ónodi:** Conceptualization (supporting); investigation (lead); methodology (supporting); project administration (supporting); visualization (supporting); writing – review and editing (lead). **Khanh Vu Ho:** Formal analysis (lead); investigation (supporting); methodology (lead); software (lead); visualization (lead); writing – original draft (supporting); writing – review and editing (lead). **Eszter Tanács:** Investigation (supporting); writing – original draft (supporting); writing – review and editing (supporting). **Rabuogi Quinter Akinyi:** Investigation (supporting); writing – original draft (supporting); writing – review and editing (lead). **Péter Török:** Funding acquisition (supporting); investigation (supporting); writing – original draft (supporting); writing – review and editing (lead). **Csaba Tölgyesi:** Investigation (supporting); writing – original draft (supporting); writing – review and editing (lead). **Zoltán Bátori:** Investigation (supporting); writing – original draft (supporting); writing – review and editing (lead). **György Kröel‐Dulay:** Conceptualization (lead); funding acquisition (lead); investigation (lead); methodology (supporting); project administration (lead); resources (lead); supervision (lead); writing – review and editing (lead).

## CONFLICT OF INTEREST STATEMENT

The authors declare that they have no known competing financial interests or personal relationships that could have appeared to influence the work reported in this paper.

## Data Availability

The data that support the findings of this study are openly available in Zenodo at https://doi.org/10.5281/zenodo.10685196.
